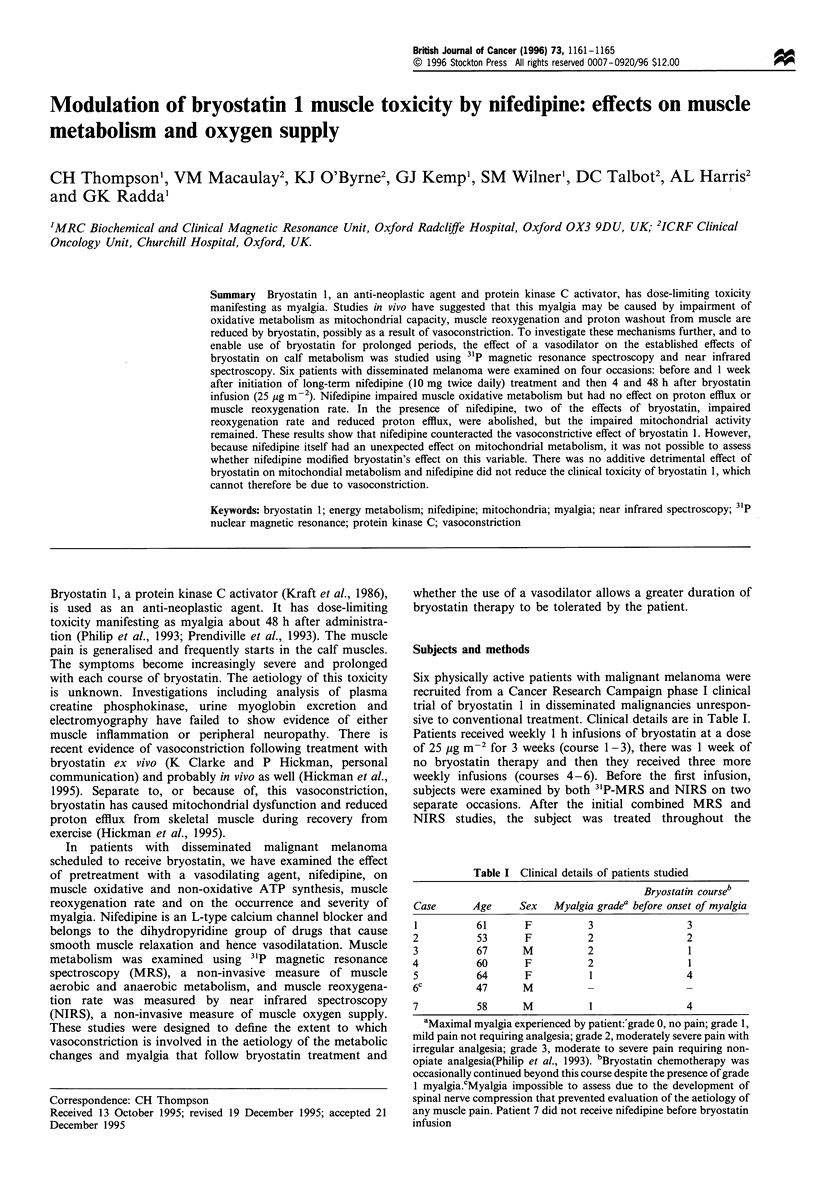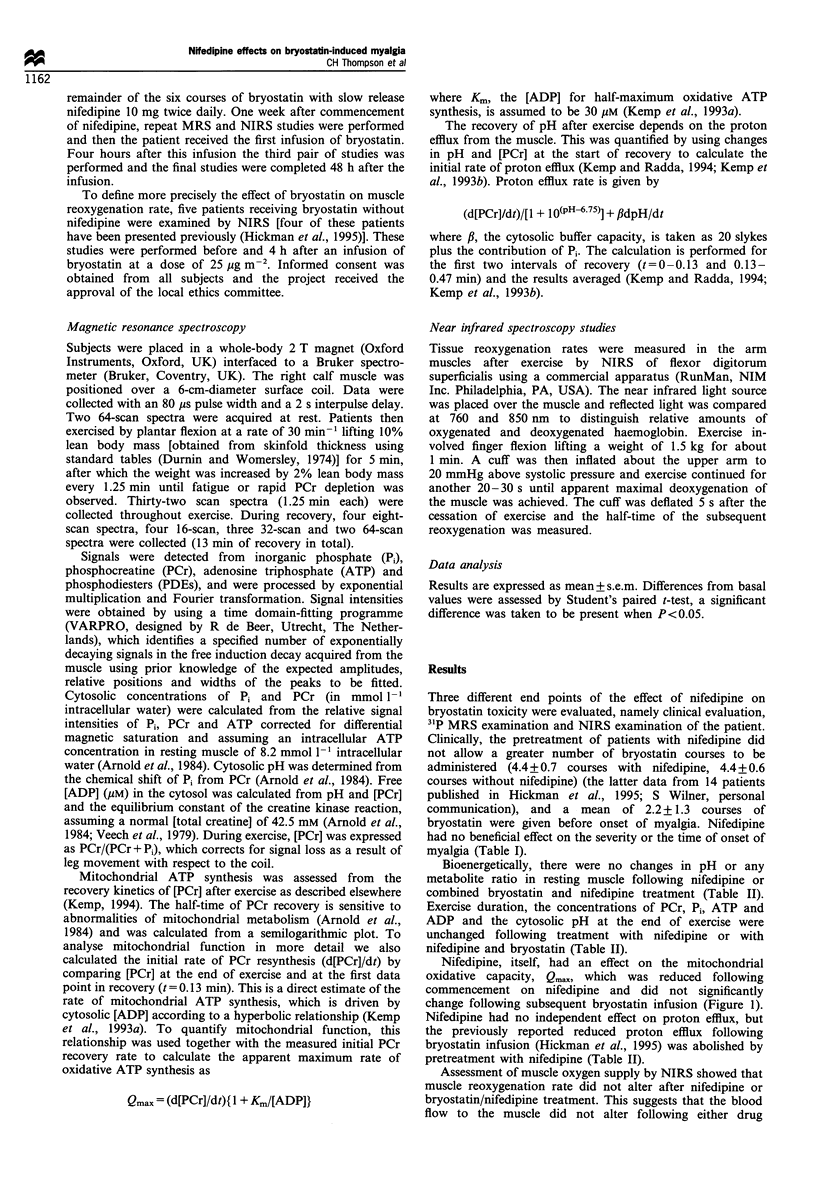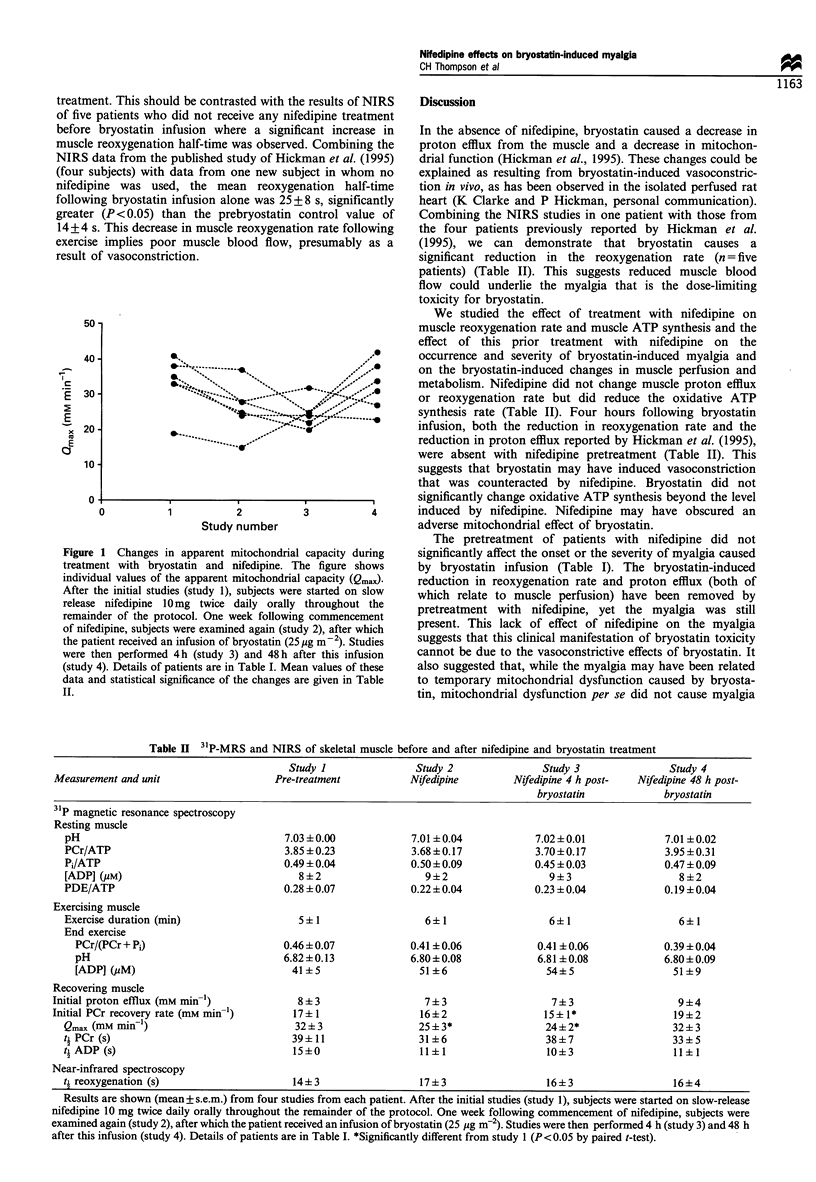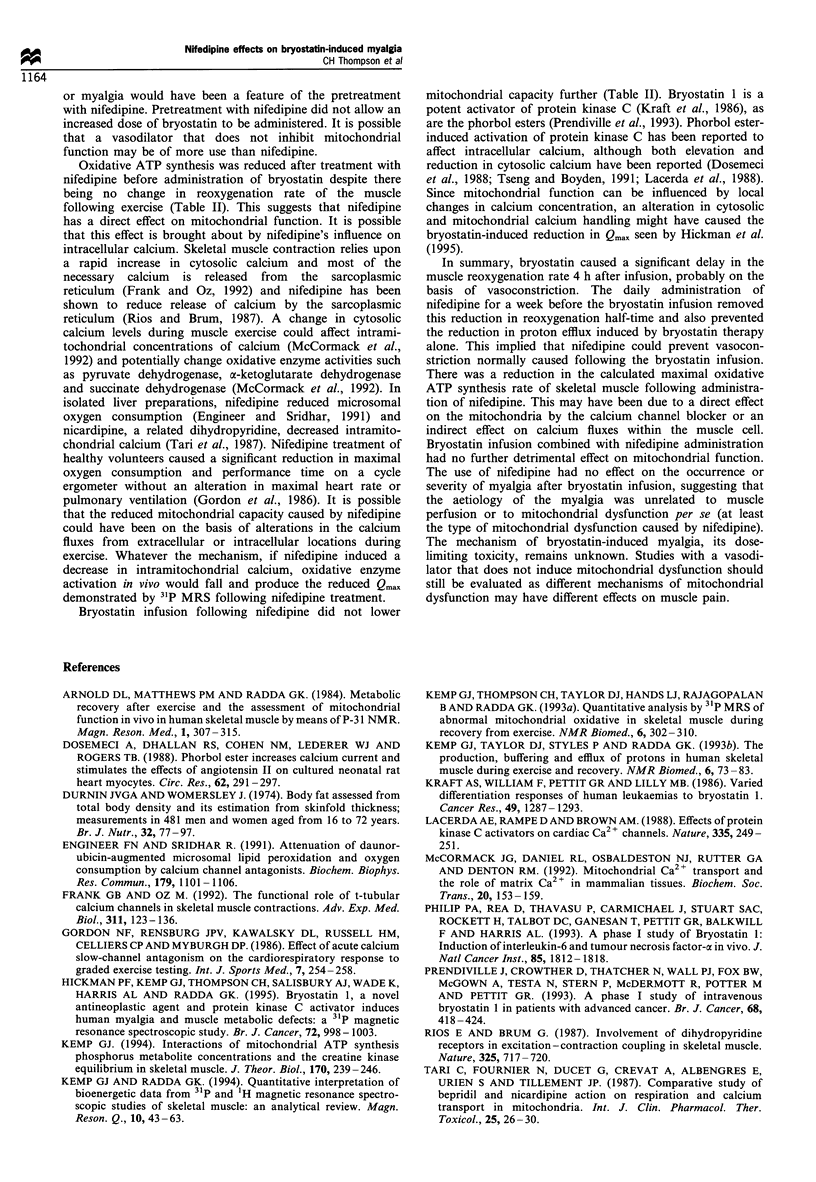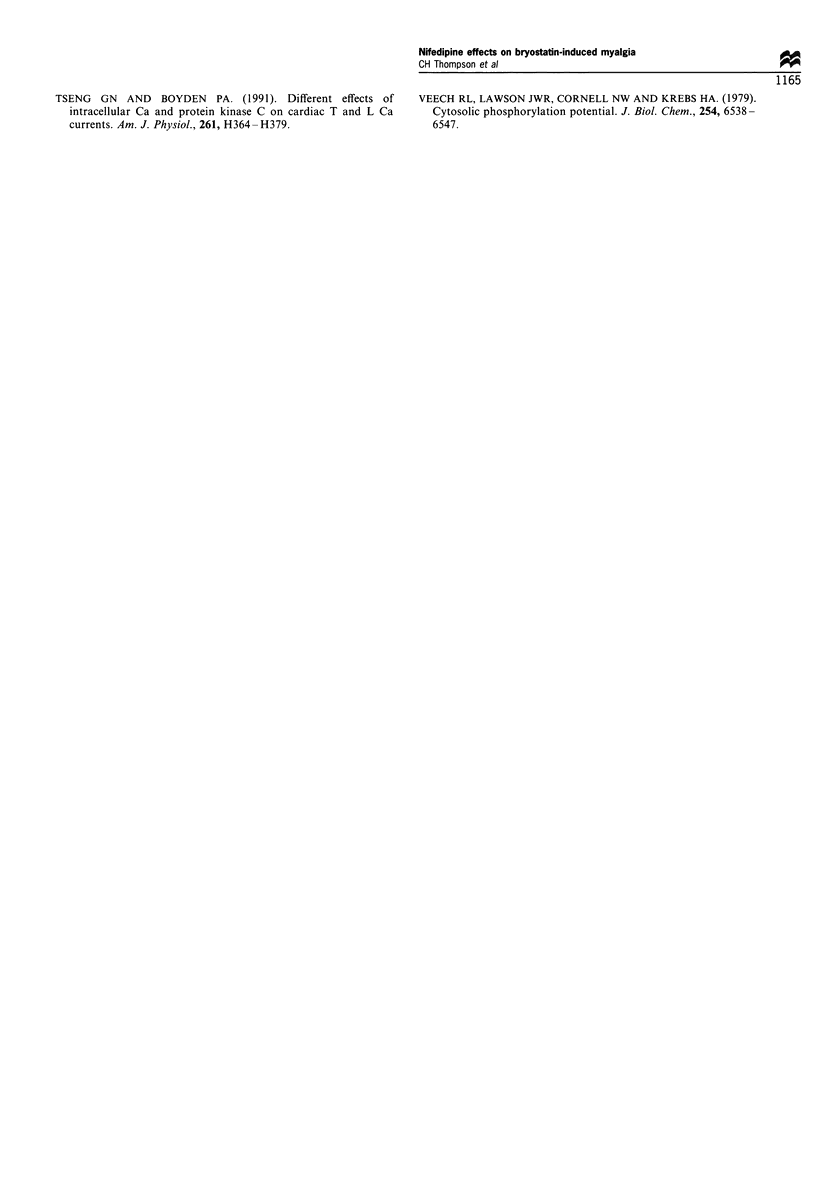# Modulation of bryostatin 1 muscle toxicity by nifedipine: effects on muscle metabolism and oxygen supply.

**DOI:** 10.1038/bjc.1996.224

**Published:** 1996-05

**Authors:** C. H. Thompson, V. M. Macaulay, K. J. O'Byrne, G. J. Kemp, S. M. Wilner, D. C. Talbot, A. L. Harris, G. K. Radda

**Affiliations:** MRC Biochemical and Clinical Magnetic Resonance Unit, Oxford Radcliffe Hospital, UK.

## Abstract

Bryostatin 1, an anti-neoplastic agent and protein kinase C activator, has dose-limiting toxicity manifesting as myalgia. Studies in vivo have suggested that this myalgia may be caused by impairment of oxidative metabolism as mitochondrial capacity, muscle reoxygenation and proton washout from muscle are reduced by bryostatin, possibly as a result of vasoconstriction. To investigate these mechanisms further, and to enable use of bryostatin for prolonged periods, the effect of a vasodilator on the established effects of bryostatin on calf metabolism was studied using 31P magnetic resonance spectroscopy and near infrared spectroscopy. Six patients with disseminated melanoma were examined on four occasions: before and 1 week after initiation of long-term nifedipine (10 mg twice daily) treatment and then 4 and 48 h after bryostatin infusion (25 micrograms m(-2)). Nifedipine impaired muscle oxidative metabolism but had no effect on proton efflux or muscle reoxygenation rate. In the presence of nifedipine, two of the effects of bryostatin, impaired reoxygenation rate and reduced proton efflux, were abolished, but the impaired mitochondrial activity remained. These results show that nifedipine counteracted the vasoconstrictive effect of bryostatin 1. However, because nifedipine itself had an unexpected effect on mitochondrial metabolism, it was not possible to assess whether nifedipine modified bryostatin's effect on this variable. There was no additive detrimental effect of bryostatin on mitochondrial metabolism and nifedipine did not reduce the clinical toxicity of bryostatin 1, which cannot therefore be due to vasoconstriction.


British Journal of Cancer (1996) 73, 1161-1165
© 1996 Stockton Press All rights reserved 0007-0920/96 $12.00

### Modulation of bryostatin 1 muscle toxicity by nifedipine: effects on muscle metabolism and oxygen supply

CH Thompson[1], VM Macaulay[2], KJ O'Byrne[2], GJ Kemp[1], SM Wilner[1], DC Talbot[2], AL Harris[2] and GK Radda[1]

[1]*MRC Biochemical and Clinical Magnetic Resonance Unit, Oxford Radcliffe Hospital, Oxford OX3 9DU, UK;* [2]*ICRF Clinical Oncology Unit, Churchill Hospital, Oxford, UK.*

**Summary** Bryostatin 1, an anti-neoplastic agent and protein kinase C activator, has dose-limiting toxicity manifesting as myalgia. Studies *in vivo* have suggested that this myalgia may be caused by impairment of oxidative metabolism as mitochondrial capacity, muscle reoxygenation and proton washout from muscle are reduced by bryostatin, possibly as a result of vasoconstriction. To investigate these mechanisms further, and to enable use of bryostatin for prolonged periods, the effect of a vasodilator on the established effects of bryostatin on calf metabolism was studied using $^{31}P$ magnetic resonance spectroscopy and near infrared spectroscopy. Six patients with disseminated melanoma were examined on four occasions: before and 1 week after initiation of long-term nifedipine (10 mg twice daily) treatment and then 4 and 48 h after bryostatin infusion (25 $\mu g\ m^{-2}$). Nifedipine impaired muscle oxidative metabolism but had no effect on proton efflux or muscle reoxygenation rate. In the presence of nifedipine, two of the effects of bryostatin, impaired reoxygenation rate and reduced proton efflux, were abolished, but the impaired mitochondrial activity remained. These results show that nifedipine counteracted the vasoconstrictive effect of bryostatin 1. However, because nifedipine itself had an unexpected effect on mitochondrial metabolism, it was not possible to assess whether nifedipine modified bryostatin's effect on this variable. There was no additive detrimental effect of bryostatin on mitochondial metabolism and nifedipine did not reduce the clinical toxicity of bryostatin 1, which cannot therefore be due to vasoconstriction.

**Keywords:** bryostatin 1; energy metabolism; nifedipine; mitochondria; myalgia; near infrared spectroscopy; $^{31}P$ nuclear magnetic resonance; protein kinase C; vasoconstriction

Bryostatin 1, a protein kinase C activator (Kraft *et al.*, 1986), is used as an anti-neoplastic agent. It has dose-limiting toxicity manifesting as myalgia about 48 h after administration (Philip *et al.*, 1993; Prendiville *et al.*, 1993). The muscle pain is generalised and frequently starts in the calf muscles. The symptoms become increasingly severe and prolonged with each course of bryostatin. The aetiology of this toxicity is unknown. Investigations including analysis of plasma creatine phosphokinase, urine myoglobin excretion and electromyography have failed to show evidence of either muscle inflammation or peripheral neuropathy. There is recent evidence of vasoconstriction following treatment with bryostatin *ex vivo* (K Clarke and P Hickman, personal communication) and probably *in vivo* as well (Hickman *et al.*, 1995). Separate to, or because of, this vasoconstriction, bryostatin has caused mitochondrial dysfunction and reduced proton efflux from skeletal muscle during recovery from exercise (Hickman *et al.*, 1995).

In patients with disseminated malignant melanoma scheduled to receive bryostatin, we have examined the effect of pretreatment with a vasodilating agent, nifedipine, on muscle oxidative and non-oxidative ATP synthesis, muscle reoxygenation rate and on the occurrence and severity of myalgia. Nifedipine is an L-type calcium channel blocker and belongs to the dihydropyridine group of drugs that cause smooth muscle relaxation and hence vasodilatation. Muscle metabolism was examined using $^{31}P$ magnetic resonance spectroscopy (MRS), a non-invasive measure of muscle aerobic and anaerobic metabolism, and muscle reoxygenation rate was measured by near infrared spectroscopy (NIRS), a non-invasive measure of muscle oxygen supply. These studies were designed to define the extent to which vasoconstriction is involved in the aetiology of the metabolic changes and myalgia that follow bryostatin treatment and whether the use of a vasodilator allows a greater duration of bryostatin therapy to be tolerated by the patient.

Correspondence: CH Thompson
Received 13 October 1995; revised 19 December 1995; accepted 21 December 1995

### Subjects and methods

Six physically active patients with malignant melanoma were recruited from a Cancer Research Campaign phase I clinical trial of bryostatin 1 in disseminated malignancies unresponsive to conventional treatment. Clinical details are in Table I. Patients received weekly 1 h infusions of bryostatin at a dose of 25 $\mu g\ m^{-2}$ for 3 weeks (course 1-3), there was 1 week of no bryostatin therapy and then they received three more weekly infusions (courses 4-6). Before the first infusion, subjects were examined by both $^{31}P$-MRS and NIRS on two separate occasions. After the initial combined MRS and NIRS studies, the subject was treated throughout the

**Table I** Clinical details of patients studied

| *Case* | *Age* | *Sex* | *Myalgia grade*[a] | *Bryostatin course*[b] *before onset of myalgia* |
|---|---|---|---|---|
| 1 | 61 | F | 3 | 3 |
| 2 | 53 | F | 2 | 2 |
| 3 | 67 | M | 2 | 1 |
| 4 | 60 | F | 2 | 1 |
| 5 | 64 | F | 1 | 4 |
| 6[c] | 47 | M | – | – |
| 7 | 58 | M | 1 | 4 |

[a]Maximal myalgia experienced by patient: grade 0, no pain; grade 1, mild pain not requiring analgesia; grade 2, moderately severe pain with irregular analgesia; grade 3, moderate to severe pain requiring non-opiate analgesia(Philip *et al.*, 1993). [b]Bryostatin chemotherapy was occasionally continued beyond this course despite the presence of grade 1 myalgia.[c]Myalgia impossible to assess due to the development of spinal nerve compression that prevented evaluation of the aetiology of any muscle pain. Patient 7 did not receive nifedipine before bryostatin infusion remainder of the six courses of bryostatin with slow release nifedipine 10 mg twice daily. One week after commencement of nifedipine, repeat MRS and NIRS studies were performed and then the patient received the first infusion of bryostatin. Four hours after this infusion the third pair of studies was performed and the final studies were completed 48 h after the infusion.

To define more precisely the effect of bryostatin on muscle reoxygenation rate, five patients receiving bryostatin without nifedipine were examined by NIRS [four of these patients have been presented previously (Hickman *et al.*, 1995)]. These studies were performed before and 4 h after an infusion of bryostatin at a dose of 25 $\mu g\ m^{-2}$. Informed consent was obtained from all subjects and the project received the approval of the local ethics committee.

*Magnetic resonance spectroscopy*

Subjects were placed in a whole-body 2 T magnet (Oxford Instruments, Oxford, UK) interfaced to a Bruker spectrometer (Bruker, Coventry, UK). The right calf muscle was positioned over a 6-cm-diameter surface coil. Data were collected with an 80 $\mu s$ pulse width and a 2 s interpulse delay. Two 64-scan spectra were acquired at rest. Patients then exercised by plantar flexion at a rate of 30 $min^{-1}$ lifting 10% lean body mass [obtained from skinfold thickness using standard tables (Durnin and Womersley, 1974)] for 5 min, after which the weight was increased by 2% lean body mass every 1.25 min until fatigue or rapid PCr depletion was observed. Thirty-two scan spectra (1.25 min each) were collected throughout exercise. During recovery, four eight-scan spectra, four 16-scan, three 32-scan and two 64-scan spectra were collected (13 min of recovery in total).

Signals were detected from inorganic phosphate ($P_i$), phosphocreatine (PCr), adenosine triphosphate (ATP) and phosphodiesters (PDEs), and were processed by exponential multiplication and Fourier transformation. Signal intensities were obtained by using a time domain-fitting programme (VARPRO, designed by R de Beer, Utrecht, The Netherlands), which identifies a specified number of exponentially decaying signals in the free induction decay acquired from the muscle using prior knowledge of the expected amplitudes, relative positions and widths of the peaks to be fitted. Cytosolic concentrations of $P_i$ and PCr (in mmol $l^{-1}$ intracellular water) were calculated from the relative signal intensities of $P_i$, PCr and ATP corrected for differential magnetic saturation and assuming an intracellular ATP concentration in resting muscle of 8.2 mmol $l^{-1}$ intracellular water (Arnold *et al.*, 1984). Cytosolic pH was determined from the chemical shift of $P_i$ from PCr (Arnold *et al.*, 1984). Free [ADP] ($\mu$M) in the cytosol was calculated from pH and [PCr] and the equilibrium constant of the creatine kinase reaction, assuming a normal [total creatine] of 42.5 mM (Arnold *et al.*, 1984; Veech *et al.*, 1979). During exercise, [PCr] was expressed as PCr/(PCr + $P_i$), which corrects for signal loss as a result of leg movement with respect to the coil.

Mitochondrial ATP synthesis was assessed from the recovery kinetics of [PCr] after exercise as described elsewhere (Kemp, 1994). The half-time of PCr recovery is sensitive to abnormalities of mitochondrial metabolism (Arnold *et al.*, 1984) and was calculated from a semilogarithmic plot. To analyse mitochondrial function in more detail we also calculated the initial rate of PCr resynthesis (d[PCr]/d$t$) by comparing [PCr] at the end of exercise and at the first data point in recovery ($t$ = 0.13 min). This is a direct estimate of the rate of mitochondrial ATP synthesis, which is driven by cytosolic [ADP] according to a hyperbolic relationship (Kemp *et al.*, 1993*a*). To quantify mitochondrial function, this relationship was used together with the measured initial PCr recovery rate to calculate the apparent maximum rate of oxidative ATP synthesis as

$$Q_{max} = (d[PCr]/dt)\{1 + K_m/[ADP]\}$$

where $K_m$, the [ADP] for half-maximum oxidative ATP synthesis, is assumed to be 30 $\mu$M (Kemp *et al.*, 1993*a*).

The recovery of pH after exercise depends on the proton efflux from the muscle. This was quantified by using changes in pH and [PCr] at the start of recovery to calculate the initial rate of proton efflux (Kemp and Radda, 1994; Kemp *et al.*, 1993*b*). Proton efflux rate is given by

$$(d[PCr]/dt)/[1 + 10^{(pH-6.75)}] + \beta dpH/dt$$

where $\beta$, the cytosolic buffer capacity, is taken as 20 slykes plus the contribution of $P_i$. The calculation is performed for the first two intervals of recovery ($t$ = 0–0.13 and 0.13–0.47 min) and the results averaged (Kemp and Radda, 1994; Kemp *et al.*, 1993*b*).

*Near infrared spectroscopy studies*

Tissue reoxygenation rates were measured in the arm muscles after exercise by NIRS of flexor digitorum superficialis using a commercial apparatus (RunMan, NIM Inc. Philadelphia, PA, USA). The near infrared light source was placed over the muscle and reflected light was compared at 760 and 850 nm to distinguish relative amounts of oxygenated and deoxygenated haemoglobin. Exercise involved finger flexion lifting a weight of 1.5 kg for about 1 min. A cuff was then inflated about the upper arm to 20 mmHg above systolic pressure and exercise continued for another 20–30 s until apparent maximal deoxygenation of the muscle was achieved. The cuff was deflated 5 s after the cessation of exercise and the half-time of the subsequent reoxygenation was measured.

*Data analysis*

Results are expressed as mean ± s.e.m. Differences from basal values were assessed by Student's paired $t$-test, a significant difference was taken to be present when $P < 0.05$.

### Results

Three different end points of the effect of nifedipine on bryostatin toxicity were evaluated, namely clinical evaluation, $^{31}P$ MRS examination and NIRS examination of the patient. Clinically, the pretreatment of patients with nifedipine did not allow a greater number of bryostatin courses to be administered (4.4 ± 0.7 courses with nifedipine, 4.4 ± 0.6 courses without nifedipine) (the latter data from 14 patients published in Hickman *et al.*, 1995; S Wilner, personal communication), and a mean of 2.2 ± 1.3 courses of bryostatin were given before onset of myalgia. Nifedipine had no beneficial effect on the severity or the time of onset of myalgia (Table I).

Bioenergetically, there were no changes in pH or any metabolite ratio in resting muscle following nifedipine or combined bryostatin and nifedipine treatment (Table II). Exercise duration, the concentrations of PCr, $P_i$, ATP and ADP and the cytosolic pH at the end of exercise were unchanged following treatment with nifedipine or with nifedipine and bryostatin (Table II).

Nifedipine, itself, had an effect on the mitochondrial oxidative capacity, $Q_{max}$, which was reduced following commencement on nifedipine and did not significantly change following subsequent bryostatin infusion (Figure 1). Nifedipine had no independent effect on proton efflux, but the previously reported reduced proton efflux following bryostatin infusion (Hickman *et al.*, 1995) was abolished by pretreatment with nifedipine (Table II).

Assessment of muscle oxygen supply by NIRS showed that muscle reoxygenation rate did not alter after nifedipine or bryostatin/nifedipine treatment. This suggests that the blood flow to the muscle did not alter following either drug treatment. This should be contrasted with the results of NIRS of five patients who did not receive any nifedipine treatment before bryostatin infusion where a significant increase in muscle reoxygenation half-time was observed. Combining the NIRS data from the published study of Hickman *et al.* (1995) (four subjects) with data from one new subject in whom no nifedipine was used, the mean reoxygenation half-time following bryostatin infusion alone was $25 \pm 8$ s, significantly greater ($P<0.05$) than the prebryostatin control value of $14 \pm 4$ s. This decrease in muscle reoxygenation rate following exercise implies poor muscle blood flow, presumably as a result of vasoconstriction.

**Figure 1** Changes in apparent mitochondrial capacity during treatment with bryostatin and nifedipine. The figure shows individual values of the apparent mitochondrial capacity ($Q_{max}$). After the initial studies (study 1), subjects were started on slow release nifedipine 10 mg twice daily orally throughout the remainder of the protocol. One week following commencement of nifedipine, subjects were examined again (study 2), after which the patient received an infusion of bryostatin (25 $\mu$g m$^{-2}$). Studies were then performed 4 h (study 3) and 48 h after this infusion (study 4). Details of patients are in Table I. Mean values of these data and statistical significance of the changes are given in Table II.

### Discussion

In the absence of nifedipine, bryostatin caused a decrease in proton efflux from the muscle and a decrease in mitochondrial function (Hickman *et al.*, 1995). These changes could be explained as resulting from bryostatin-induced vasoconstriction *in vivo*, as has been observed in the isolated perfused rat heart (K Clarke and P Hickman, personal communication). Combining the NIRS studies in one patient with those from the four patients previously reported by Hickman *et al.* (1995), we can demonstrate that bryostatin causes a significant reduction in the reoxygenation rate ($n$ = five patients) (Table II). This suggests reduced muscle blood flow could underlie the myalgia that is the dose-limiting toxicity for bryostatin.

We studied the effect of treatment with nifedipine on muscle reoxygenation rate and muscle ATP synthesis and the effect of this prior treatment with nifedipine on the occurrence and severity of bryostatin-induced myalgia and on the bryostatin-induced changes in muscle perfusion and metabolism. Nifedipine did not change muscle proton efflux or reoxygenation rate but did reduce the oxidative ATP synthesis rate (Table II). Four hours following bryostatin infusion, both the reduction in reoxygenation rate and the reduction in proton efflux reported by Hickman *et al.* (1995), were absent with nifedipine pretreatment (Table II). This suggests that bryostatin may have induced vasoconstriction that was counteracted by nifedipine. Bryostatin did not significantly change oxidative ATP synthesis beyond the level induced by nifedipine. Nifedipine may have obscured an adverse mitochondrial effect of bryostatin.

The pretreatment of patients with nifedipine did not significantly affect the onset or the severity of myalgia caused by bryostatin infusion (Table I). The bryostatin-induced reduction in reoxygenation rate and proton efflux (both of which relate to muscle perfusion) have been removed by pretreatment with nifedipine, yet the myalgia was still present. This lack of effect of nifedipine on the myalgia suggests that this clinical manifestation of bryostatin toxicity cannot be due to the vasoconstrictive effects of bryostatin. It also suggested that, while the myalgia may have been related to temporary mitochondrial dysfunction caused by bryostatin, mitochondrial dysfunction *per se* did not cause myalgia

**Table II** $^{31}$P-MRS and NIRS of skeletal muscle before and after nifedipine and bryostatin treatment

| *Measurement and unit* | *Study 1 Pre-treatment* | *Study 2 Nifedipine* | *Study 3 Nifedipine 4 h post-bryostatin* | *Study 4 Nifedipine 48 h post-bryostatin* |
|---|---|---|---|---|
| $^{31}$P magnetic resonance spectroscopy | | | | |
| Resting muscle | | | | |
| pH | 7.03±0.00 | 7.01±0.04 | 7.02±0.01 | 7.01±0.02 |
| PCr/ATP | 3.85±0.23 | 3.68±0.17 | 3.70±0.17 | 3.95±0.31 |
| $P_i$/ATP | 0.49±0.04 | 0.50±0.09 | 0.45±0.03 | 0.47±0.09 |
| [ADP] ($\mu$M) | 8±2 | 9±2 | 9±3 | 8±2 |
| PDE/ATP | 0.28±0.07 | 0.22±0.04 | 0.23±0.04 | 0.19±0.04 |
| Exercising muscle | | | | |
| Exercise duration (min) | 5±1 | 6±1 | 6±1 | 6±1 |
| End exercise | | | | |
| PCr/(PCr + $P_i$) | 0.46±0.07 | 0.41±0.06 | 0.41±0.06 | 0.39±0.04 |
| pH | 6.82±0.13 | 6.80±0.08 | 6.81±0.08 | 6.80±0.09 |
| [ADP] ($\mu$M) | 41±5 | 51±6 | 54±5 | 51±9 |
| Recovering muscle | | | | |
| Initial proton efflux (mM min$^{-1}$) | 8±3 | 7±3 | 7±3 | 9±4 |
| Initial PCr recovery rate (mM min$^{-1}$) | 17±1 | 16±2 | 15±1* | 19±2 |
| $Q_{max}$ (mM min$^{-1}$) | 32±3 | 25±3* | 24±2* | 32±3 |
| $t_{1/2}$ PCr (s) | 39±11 | 31±6 | 38±7 | 33±5 |
| $t_{1/2}$ ADP (s) | 15±0 | 11±1 | 10±3 | 11±1 |
| Near-infrared spectroscopy | | | | |
| $t_{1/2}$ reoxygenation (s) | 14±3 | 17±3 | 16±3 | 16±4 |

Results are shown (mean±s.e.m.) from four studies from each patient. After the initial studies (study 1), subjects were started on slow-release nifedipine 10 mg twice daily orally throughout the remainder of the protocol. One week following commencement of nifedipine, subjects were examined again (study 2), after which the patient received an infusion of bryostatin (25 $\mu$g m$^{-2}$). Studies were then performed 4 h (study 3) and 48 h after this infusion (study 4). Details of patients are in Table I. *Significantly different from study 1 ($P<0.05$ by paired $t$-test).

or myalgia would have been a feature of the pretreatment with nifedipine. Pretreatment with nifedipine did not allow an increased dose of bryostatin to be administered. It is possible that a vasodilator that does not inhibit mitochondrial function may be of more use than nifedipine.

Oxidative ATP synthesis was reduced after treatment with nifedipine before administration of bryostatin despite there being no change in reoxygenation rate of the muscle following exercise (Table II). This suggests that nifedipine has a direct effect on mitochondrial function. It is possible that this effect is brought about by nifedipine's influence on intracellular calcium. Skeletal muscle contraction relies upon a rapid increase in cytosolic calcium and most of the necessary calcium is released from the sarcoplasmic reticulum (Frank and Oz, 1992) and nifedipine has been shown to reduce release of calcium by the sarcoplasmic reticulum (Rios and Brum, 1987). A change in cytosolic calcium levels during muscle exercise could affect intramitochondrial concentrations of calcium (McCormack *et al.*, 1992) and potentially change oxidative enzyme activities such as pyruvate dehydrogenase, α-ketoglutarate dehydrogenase and succinate dehydrogenase (McCormack *et al.*, 1992). In isolated liver preparations, nifedipine reduced microsomal oxygen consumption (Engineer and Sridhar, 1991) and nicardipine, a related dihydropyridine, decreased intramitochondrial calcium (Tari *et al.*, 1987). Nifedipine treatment of healthy volunteers caused a significant reduction in maximal oxygen consumption and performance time on a cycle ergometer without an alteration in maximal heart rate or pulmonary ventilation (Gordon *et al.*, 1986). It is possible that the reduced mitochondrial capacity caused by nifedipine could have been on the basis of alterations in the calcium fluxes from extracellular or intracellular locations during exercise. Whatever the mechanism, if nifedipine induced a decrease in intramitochondrial calcium, oxidative enzyme activation *in vivo* would fall and produce the reduced $Q_{max}$ demonstrated by $^{31}P$ MRS following nifedipine treatment.

Bryostatin infusion following nifedipine did not lower mitochondrial capacity further (Table II). Bryostatin 1 is a potent activator of protein kinase C (Kraft *et al.*, 1986), as are the phorbol esters (Prendiville *et al.*, 1993). Phorbol ester-induced activation of protein kinase C has been reported to affect intracellular calcium, although both elevation and reduction in cytosolic calcium have been reported (Dosemeci *et al.*, 1988; Tseng and Boyden, 1991; Lacerda *et al.*, 1988). Since mitochondrial function can be influenced by local changes in calcium concentration, an alteration in cytosolic and mitochondrial calcium handling might have caused the bryostatin-induced reduction in $Q_{max}$ seen by Hickman *et al.* (1995).

In summary, bryostatin caused a significant delay in the muscle reoxygenation rate 4 h after infusion, probably on the basis of vasoconstriction. The daily administration of nifedipine for a week before the bryostatin infusion removed this reduction in reoxygenation half-time and also prevented the reduction in proton efflux induced by bryostatin therapy alone. This implied that nifedipine could prevent vasoconstriction normally caused following the bryostatin infusion. There was a reduction in the calculated maximal oxidative ATP synthesis rate of skeletal muscle following administration of nifedipine. This may have been due to a direct effect on the mitochondria by the calcium channel blocker or an indirect effect on calcium fluxes within the muscle cell. Bryostatin infusion combined with nifedipine administration had no further detrimental effect on mitochondrial function. The use of nifedipine had no effect on the occurrence or severity of myalgia after bryostatin infusion, suggesting that the aetiology of the myalgia was unrelated to muscle perfusion or to mitochondrial dysfunction *per se* (at least the type of mitochondrial dysfunction caused by nifedipine). The mechanism of bryostatin-induced myalgia, its dose-limiting toxicity, remains unknown. Studies with a vasodilator that does not induce mitochondrial dysfunction should still be evaluated as different mechanisms of mitochondrial dysfunction may have different effects on muscle pain.

### References

ARNOLD DL, MATTHEWS PM AND RADDA GK. (1984). Metabolic recovery after exercise and the assessment of mitochondrial function in vivo in human skeletal muscle by means of P-31 NMR. *Magn. Reson. Med.*, **1**, 307–315.

DOSEMECI A, DHALLAN RS, COHEN NM, LEDERER WJ AND ROGERS TB. (1988). Phorbol ester increases calcium current and stimulates the effects of angiotensin II on cultured neonatal rat heart myocytes. *Circ. Res.*, **62**, 291–297.

DURNIN JVGA AND WOMERSLEY J. (1974). Body fat assessed from total body density and its estimation from skinfold thickness; measurements in 481 men and women aged from 16 to 72 years. *Br. J. Nutr.*, **32**, 77–97.

ENGINEER FN AND SRIDHAR R. (1991). Attenuation of daunorubicin-augmented microsomal lipid peroxidation and oxygen consumption by calcium channel antagonists. *Biochem. Biophys. Res. Commun.*, **179**, 1101–1106.

FRANK GB AND OZ M. (1992). The functional role of t-tubular calcium channels in skeletal muscle contractions. *Adv. Exp. Med. Biol.*, **311**, 123–136.

GORDON NF, RENSBURG JPV, KAWALSKY DL, RUSSELL HM, CELLIERS CP AND MYBURGH DP. (1986). Effect of acute calcium slow-channel antagonism on the cardiorespiratory response to graded exercise testing. *Int. J. Sports Med.*, **7**, 254–258.

HICKMAN PF, KEMP GJ, THOMPSON CH, SALISBURY AJ, WADE K, HARRIS AL AND RADDA GK. (1995). Bryostatin 1, a novel antineoplastic agent and protein kinase C activator induces human myalgia and muscle metabolic defects: a $^{31}P$ magnetic resonance spectroscopic study. *Br. J. Cancer*, **72**, 998–1003.

KEMP GJ. (1994). Interactions of mitochondrial ATP synthesis phosphorus metabolite concentrations and the creatine kinase equilibrium in skeletal muscle. *J. Theor. Biol.*, **170**, 239–246.

KEMP GJ AND RADDA GK. (1994). Quantitative interpretation of bioenergetic data from $^{31}P$ and $^{1}H$ magnetic resonance spectroscopic studies of skeletal muscle: an analytical review. *Magn. Reson. Q.*, **10**, 43–63.

KEMP GJ, THOMPSON CH, TAYLOR DJ, HANDS LJ, RAJAGOPALAN B AND RADDA GK. (1993*a*). Quantitative analysis by $^{31}P$ MRS of abnormal mitochondrial oxidative in skeletal muscle during recovery from exercise. *NMR Biomed.*, **6**, 302–310.

KEMP GJ, TAYLOR DJ, STYLES P AND RADDA GK. (1993*b*). The production, buffering and efflux of protons in human skeletal muscle during exercise and recovery. *NMR Biomed.*, **6**, 73–83.

KRAFT AS, WILLIAM F, PETTIT GR AND LILLY MB. (1986). Varied differentiation responses of human leukaemias to bryostatin 1. *Cancer Res.*, **49**, 1287–1293.

LACERDA AE, RAMPE D AND BROWN AM. (1988). Effects of protein kinase C activators on cardiac $Ca^{2+}$ channels. *Nature*, **335**, 249–251.

McCORMACK JG, DANIEL RL, OSBALDESTON NJ, RUTTER GA AND DENTON RM. (1992). Mitochondrial $Ca^{2+}$ transport and the role of matrix $Ca^{2+}$ in mammalian tissues. *Biochem. Soc. Trans.*, **20**, 153–159.

PHILIP PA, REA D, THAVASU P, CARMICHAEL J, STUART SAC, ROCKETT H, TALBOT DC, GANESAN T, PETTIT GR, BALKWILL F AND HARRIS AL. (1993). A phase I study of Bryostatin 1: Induction of interleukin-6 and tumour necrosis factor-α in vivo. *J. Natl Cancer Inst.*, **85**, 1812–1818.

PRENDIVILLE J, CROWTHER D, THATCHER N, WALL PJ, FOX BW, McGOWN A, TESTA N, STERN P, McDERMOTT R, POTTER M AND PETTIT GR. (1993). A phase I study of intravenous bryostatin 1 in patients with advanced cancer. *Br. J. Cancer*, **68**, 418–424.

RIOS E AND BRUM G. (1987). Involvement of dihydropyridine receptors in excitation–contraction coupling in skeletal muscle. *Nature*, **325**, 717–720.

TARI C, FOURNIER N, DUCET G, CREVAT A, ALBENGRES E, URIEN S AND TILLEMENT JP. (1987). Comparative study of bepridil and nicardipine action on respiration and calcium transport in mitochondria. *Int. J. Clin. Pharmacol. Ther. Toxicol.*, **25**, 26–30.

TSENG GN AND BOYDEN PA. (1991). Different effects of intracellular Ca and protein kinase C on cardiac T and L Ca currents. *Am. J. Physiol.*, **261,** H364–H379.

VEECH RL, LAWSON JWR, CORNELL NW AND KREBS HA. (1979). Cytosolic phosphorylation potential. *J. Biol. Chem.*, **254,** 6538–6547.